# Increased Monocyte Turnover from Bone Marrow Correlates with Severity of SIV Encephalitis and CD163 Levels in Plasma

**DOI:** 10.1371/journal.ppat.1000842

**Published:** 2010-04-15

**Authors:** Tricia H. Burdo, Caroline Soulas, Krystyna Orzechowski, Jessica Button, Anitha Krishnan, Chie Sugimoto, Xavier Alvarez, Marcelo J. Kuroda, Kenneth C. Williams

**Affiliations:** 1 Biology Department, Boston College, Chestnut Hill, Massachusetts, United States of America; 2 Division of Immunology, Tulane National Primate Research Center, Tulane University Health Science Center, Covington, Louisiana, United States of America; 3 Division of Comparative Pathology, Tulane National Primate Research Center, Tulane University Health Science Center, Covington, Louisiana, United States of America; NIH/NIAID, United States of America

## Abstract

Cells of the myeloid lineage are significant targets for human immunodeficiency virus (HIV) in humans and simian immunodeficiency virus (SIV) in monkeys. Monocytes play critical roles in innate and adaptive immunity during inflammation. We hypothesize that specific subsets of monocytes expand with AIDS and drive central nervous system (CNS) disease. Additionally, there may be expansion of cells from the bone marrow through blood with subsequent macrophage accumulation in tissues driving pathogenesis. To identify monocytes that recently emigrated from bone marrow, we used 5-bromo-2′-deoxyuridine (BrdU) labeling in a longitudinal study of SIV-infected CD8+ T lymphocyte depleted macaques. Monocyte expansion and kinetics in blood was assessed and newly migrated monocyte/macrophages were identified within the CNS. Five animals developed rapid AIDS with differing severity of SIVE. The percentages of BrdU+ monocytes in these animals increased dramatically, early after infection, peaking at necropsy where the percentage of BrdU+ monocytes correlated with the severity of SIVE. Early analysis revealed changes in the percentages of BrdU+ monocytes between slow and rapid progressors as early as 8 days and consistently by 27 days post infection. Soluble CD163 (sCD163) in plasma correlated with the percentage of BrdU+ monocytes in blood, demonstrating a relationship between monocyte activation and expansion with disease. BrdU+ monocytes/macrophages were found within perivascular spaces and SIVE lesions. The majority (80–90%) of the BrdU+ cells were Mac387+ that were not productively infected. There was a minor population of CD68+BrdU+ cells (<10%), very few of which were infected (<1% of total BrdU+ cells). Our results suggest that an increased rate of monocyte recruitment from bone marrow into the blood correlates with rapid progression to AIDS, and the magnitude of BrdU+ monocytes correlates with the severity of SIVE.

## Author Summary

Human immunodeficiency virus (HIV) and the closely related simian immunodeficiency virus (SIV) can infect monocyte/macrophages, which enter and accumulate in the brain leading to neuronal dysfunction. Monocyte/macrophages exit the bone marrow, transit through the blood and enter the central nervous system (CNS). What triggers these cells to traffic is undefined, but it occurs in normal non-infected conditions at a rate that is accelerated with viral infection. Here, we used 5-bromo-2′-deoxyuridine (BrdU) injection and incorporation into the DNA of monocytes prior to their departure from the bone marrow. We found that the percentage of BrdU+ monocytes leaving the bone marrow 24 hours after injection increased in animals that rapidly succumbed to AIDS and correlated with the severity of SIV encephalitis (SIVE). Differences in BrdU labeled monocytes in slow and rapid progressors were revealed as early as 8 days and were consistent by 27 days post infection. Soluble CD163, shed by activated monocyte/macrophages, directly correlated with BrdU+ monocyte expansion. Our study provides new insights into the development of HIV-related CNS disease and underscores the importance of monocyte/macrophage recruitment from the bone marrow as an AIDS defining event.

## Introduction

Monocytes of bone marrow origin are circulating precursors that give rise to and replenish macrophage populations in tissues, including the brain [1]. Monocytes that originate from hematopoietic stem cells in bone marrow undergo three stages of differentiation from monoblasts to promonocytes and then monocytes where they are released into the circulation [2]–[4]. Current dogma defines that human and non-human primate monocytes do not divide out of the bone marrow [5]. Blood monocytes are thought to circulate in the vasculature for approximately 24–72 hours before differentiation into macrophages in tissues [2], [6]. Continuous extravasation and differentiation of circulating monocytic precursors has long been considered the sole source of tissue macrophages [7]. Other mechanisms to maintain tissue macrophage homeostasis have been identified and described in rodents including: 1) self-renewal of differentiated resident cells and 2) homing and limited proliferation of bone marrow derived precursors in tissues [5], [8]–[11]. Such mechanisms are not thought to function in humans. Nevertheless, in both rodents and primates in acute inflammation, monocytes are recruited to tissue compartments [2], [12]–[14]. With acute inflammation, the half-life of circulating monocytes is decreased coincident with an accumulation of macrophages at the inflamed site [15], [16]. The half-life of circulating monocytes in chronic inflammation is undefined. Prior studies of monocyte kinetics used autoradiographic analysis and radiolabeled thymidine or indium chloride incorporation, which was powerful but of limited utility due to the toxicity of radiolabel [6], [15]–[18]. More recently, we and others used the thymidine analog 5′-bromo-2′-deoxyuridine (BrdU) to quantify the turnover and release of monocytes from bone marrow [19]–[24]. BrdU is incorporated into cellular DNA during replication, at the S-phase of the cell cycle. Monocytes are released from the bone marrow into the circulation shortly after the completion of S phase, thus BrdU is a reliable marker for monocytes newly released into blood [20], [21]. We have shown that increased BrdU incorporation in monocytes with SIV infection is associated with macrophage cell death in lymph nodes [20]. Increased percentage of BrdU+ monocytes correlated with AIDS more so than CD4+ T lymphocyte loss or viral load [20]. In this report, we confirm and extend these observations in a serial pathogenesis study of monocyte expansion with emphasis on the severity of SIVE and identification of a plasma marker of monocyte expansion.

Monocytes have been shown to play a critical role in HIV and SIV disease pathogenesis [25]–[29]. The expansion of the number and/or relative percentage of CD14+CD16+ monocytes correlates with the incidence of HIV encephalitis (HIVE) and accumulation of monocyte/macrophages in HIVE lesions correlates with dementia [25], [30], [31]. Similarly, our laboratory has shown that SIV-infected CD8+ T lymphocyte depleted rhesus macaques have a biphasic increase in the percentage and absolute numbers of CD14+CD16+ monocytes with viremia and later with the development of AIDS [32]–[35]. Whether this is from increased recruitment of monocytes from bone marrow or recirculation from tissue sources is not defined.

We hypothesized that increased monocyte production from bone marrow and traffic into the brain during SIV infection correlates with rapid development of AIDS and severity of SIVE. To test this, we examined BrdU+ incorporation of monocytes in a longitudinal serial-sample pathogenesis study using SIV-infected CD8+ T lymphocyte depleted rhesus monkeys [36]–[38]. We show BrdU+ monocytes are negative for the proliferation marker Ki-67, consistent with monocytes labeled with BrdU in the marrow that do not proliferate in the blood. We demonstrate a significant correlation between the increased percentage of BrdU+ monocytes in blood at necropsy and the severity of SIV disease. Moreover, we find within CD8+ T lymphocyte depleted animals the magnitude of BrdU+ monocytes is equal with the rate of disease progression. We have identified BrdU+ monocyte/macrophages accumulating in the CNS perivascular space and SIVE lesions. About 80–90% of BrdU+ cells are Mac387+ that are not productively infected and likely representative of recently recruited monocyte/macrophages. A rare population of CD68+ macrophages are BrdU+ and also productively infected. These results suggest that an increased number of monocytes emigrating from the bone marrow occurs with rapid progression to AIDS and correlates with the severity of SIVE at necropsy. These data further point to the traffic of BrdU+ cells into the CNS. We did not find a correlation between BrdU+ monocytes in blood and plasma LPS levels, but found a correlation with soluble CD163 (sCD163) levels in plasma, consistent with monocyte activation and stimulation of innate immunity [39]–[45]. Overall these data suggest that increased monocyte production from bone marrow, traffic to the brain, and overall monocyte activation play major roles in HIV and SIV neuropathogenesis.

## Results

In this pathogenesis study, we utilized seven SIVmac251-infected rhesus macaques that were depleted of CD8+ T lymphocytes by three injections of a CD8-specific mouse-human chimeric antibody (cM-T807) at 6, 8 and 12 days post infection. Of these, one animal was CD8+ T lymphocyte depleted for 21 days (“transiently” depleted) and six for greater than 28 days (“persistently” depleted) (Table 1). Animals were sacrificed upon the development of AIDS (criteria described in Materials and Methods). This cohort could be subdivided into slow and rapid progressors. Of the rapid progressors, five animals were sacrificed with AIDS (56, 75, 77, 89 and 92 days post infection) and four had SIV encephalitis (SIVE) (criteria described in Materials and Methods). Of the two slow progressors, one was sacrificed at 131 days post infection and the other did not develop AIDS and is still alive (Table 1).

**Table 1:** SIV CD8+ T lymphocyte depleted animals used in the study.

ANIMALS	CD8+ T LYMPHOCYTE DEPLETION STATUS	SURVIVAL (DPI)/GROUP	AVG. PLASMA VIRAL LOAD (LOG 10) (SEM)	PATHOLOGY
55-05	Persistently depleted	56/Rapid	7.95 (6.94)	Mild SIVE
244-96	Persistently depleted	77/Rapid	8.05 (7.63)	Severe SIVE
DB79	Persistently depleted	92/Rapid	7.49 (6.83)	Severe SIVE
CM07	Persistently depleted	75/Rapid	7.26 (6.75)	Mild SIVE
168-05	Persistently depleted	89/Rapid	8.15 (7.08)	AIDS noE
288-07	Persistently depleted	131/Slow	8.06 (7.49)	AIDS/CMV
186-05	Transiently depleted	Alive/Slow	7.69 (7.01)	N/A

Animal numbers, CD8 depletion status, length of survival, grouping, average viral load over the course of infection and pathology at time of death are shown in this table. Persistently depleted is defined by a CD8 T lymphocyte depletion greater than 28 days. Transiently depleted is defined by a length of CD8 T lymphocyte depletion less than 21 days. DPI = days post infection, SEM = standard error of the mean, Rapid = rapid progressors, Slow = slow progressors, SIVE = SIV encephalitis, AIDS noE = animal was sacrificed with AIDS, but did not have SIVE, CMV = cytomegalovirus, N/A = not applicable.

**Figure 1:**
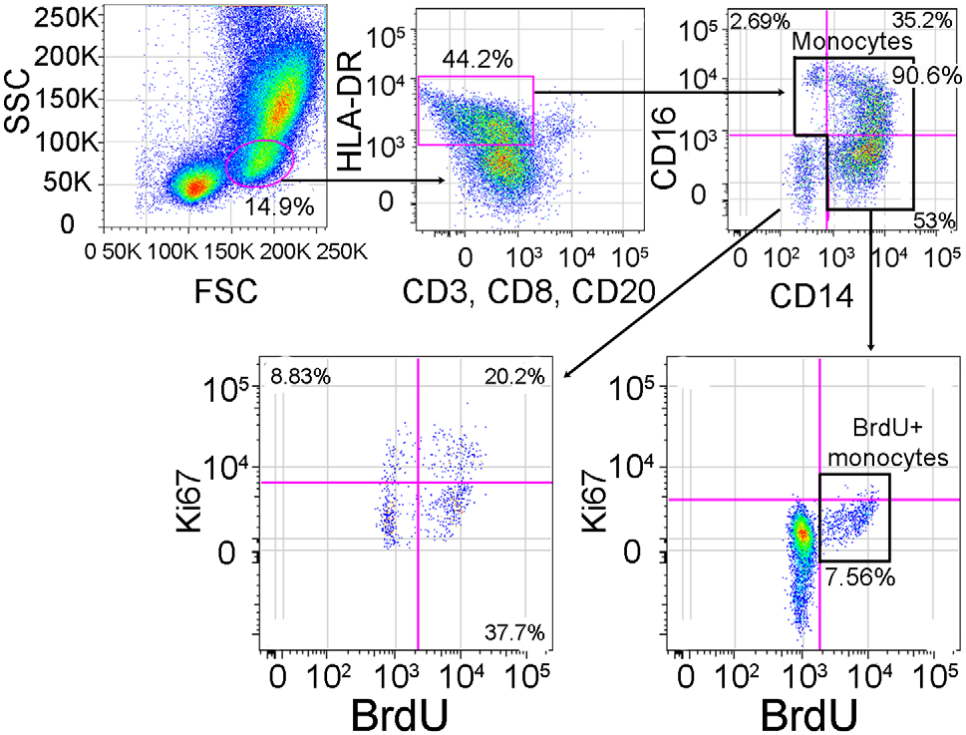
Gating strategy for identifying BrdU+ monocytes. In order to identify BrdU+ monocytes after *in vivo* BrdU injection, we used flow cytometry on whole blood. We first gated on the monocytes based on their forward (FSC) vs. side scatter (SSC) properties (far left panel), then excluded HLA-DR negative cells as well as T-lymphocytes using anti-CD3, NK cells using anti-CD8 and B-lymphocytes using anti-CD20 (second panel from left). Next, we examined monocytes based on their expression of CD14 and CD16 (right top panel). We examined the expression of Ki-67 and BrdU on monocytes (including CD14+CD16−, CD14+CD16+ and CD14−CD16+) (bottom right panel). For the purpose of this study, CD14−CD16− cells were excluded from all further analyses since this population contains CD34+ hematopoietic stem cells and dendritic cells (bottom left panel). This gating and data are representative of an infected rapid progressor at day 27 post-infection out of all animals studied.

To examine if the rate of monocyte turnover and traffic into the brain during SIV infection correlates with disease progression, we performed serial sample of monocytes 24hrs after BrdU injection at multiple time points. Flow cytometry on whole blood was used to identify BrdU+ monocytes with the gating strategy defined in Figure 1. Monocytes were first identified by FSC versus SSC profiles, and selection of HLA−DR+ cells with exclusion of CD3+, CD8+, and CD20+ cells. The percentage of BrdU+ monocytes was calculated from total monocytes: CD14+CD16−, CD14+CD16+ and CD14−CD16+ (excluding CD14−CD16− cells) (Figure 1). We have previously shown that monocytes in the bone marrow were BrdU+Ki-67+ [20]. However, in blood we found that CD14+ monocytes are BrdU+Ki-67− (Figure 1). These results are consistent with non-proliferating blood monocytes labeled with BrdU in bone marrow that lose Ki-67 post-proliferation (within 24–48 hours), when cells are in the blood [3], [46]–[49]. The CD14−CD16− cells, likely comprised of CD34+ hematopoietic stem cells and dendritic cells (DCs), are BrdU+ and Ki-67+ (Figure 1). These data are consistent with recent reports [20], [21].

**Figure 2:**
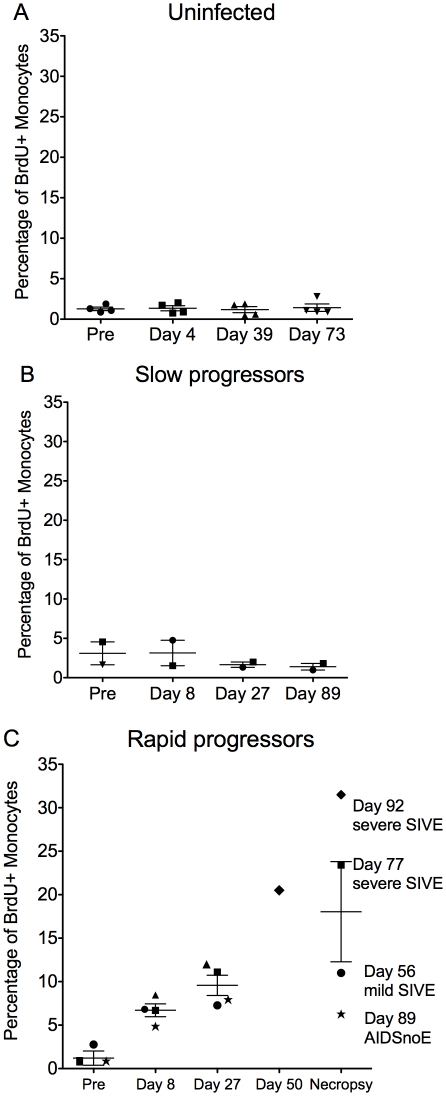
Increased percentage of BrdU+ monocytes is predictive rapid progression to AIDS and severity of SIVE. (**A–C**): BrdU was injected prior to (n = 1) and after SIVmac251 infection (n = 3) and the percentages of BrdU+ monocytes 24hrs after BrdU injection was determined by flow cytometric analysis. **A**. In four uninfected CD8+ T lymphocyte depleted animals, the percentage of BrdU+ monocytes remained approximately 2% of total monocytes in all time points. Thus, CD8+ T lymphocyte depletion alone without SIV infection does not alter monocyte turnover (n = 4). CD8+ T lymphocyte depleted SIV infected animals were divided into two groups: slow progressors (**B**) and rapid progressors (**C**). **B**. Monocyte turnover is unchanged at all points examined after infection in slow progressors (n = 2). **C**. The percentages of BrdU+ monocytes increased dramatically with rapid disease in animals that succumbed to AIDS (n = 5). The magnitude of BrdU+ incorporation of monocytes at necropsy can differentiate mild and severe SIVE. Each animal is represented by a different symbol. Error bars are standard error of the mean.

BrdU incorporation in monocytes 24hrs post-BrdU injection in control, uninfected CD8+ T lymphocyte depleted animals showed that CD8+ T lymphocyte depletion alone did not affect monocyte turnover (Figure 2A; n = 4). The percentage of BrdU+ monocytes after CD8+ T lymphocyte depletion was the same as pre-depletion (approximately 2%). There was no correlation between the percentage of BrdU+ monocytes and days post CD8+ T lymphocyte depletion in uninfected animals (r = 0.02427, P = 0.9289). There was an increase in BrdU+ monocytes in CD8+ T lymphocyte-depleted infected (Figure 2B–C) versus uninfected control animals (Figure 2A) and a higher percentage of BrdU+ monocytes in rapid (Figure 2C) versus slow progressors (Figure 2B). Differences in the percentage of BrdU+ monocytes between animals with rapid versus slow progression were evident as early as 8 and consistent at 27 days post infection (Figure 2B–C). No correlation was detected between the percentage of BrdU+ monocytes and days post infection in slow progressors (Figure 2B; r = −0.1464, P = 0.7294). In fact, the percentage of BrdU+ monocytes in these animals was similar to non-infected controls. There was a dramatic increase in the percentage of BrdU+ monocytes in the rapid progressors and a significant correlation between the percentage of BrdU+ monocytes and days post infection (Figure 2C; r = 0.7651, P = 0.0006). At end stage disease, the percentage of BrdU+ monocytes correlated with the severity of SIVE (Figure 2C). In rapid progressors, the percentage of BrdU+ cells prior to infection ranged from 0.85%, to 2.77%. This percentage increased with infection and at necropsy was 6.27% in an animal with AIDS but no SIVE (AIDS noE), 11.0% in an animal with mild SIVE, and 23.4% and 31.5% in animals with severe SIVE (Figure 2C). Thus, the percentage of BrdU+ monocytes correlated with time after infection in rapid progressors and was increased with severity of SIVE at necropsy (Figure 2C).

**Figure 3:**
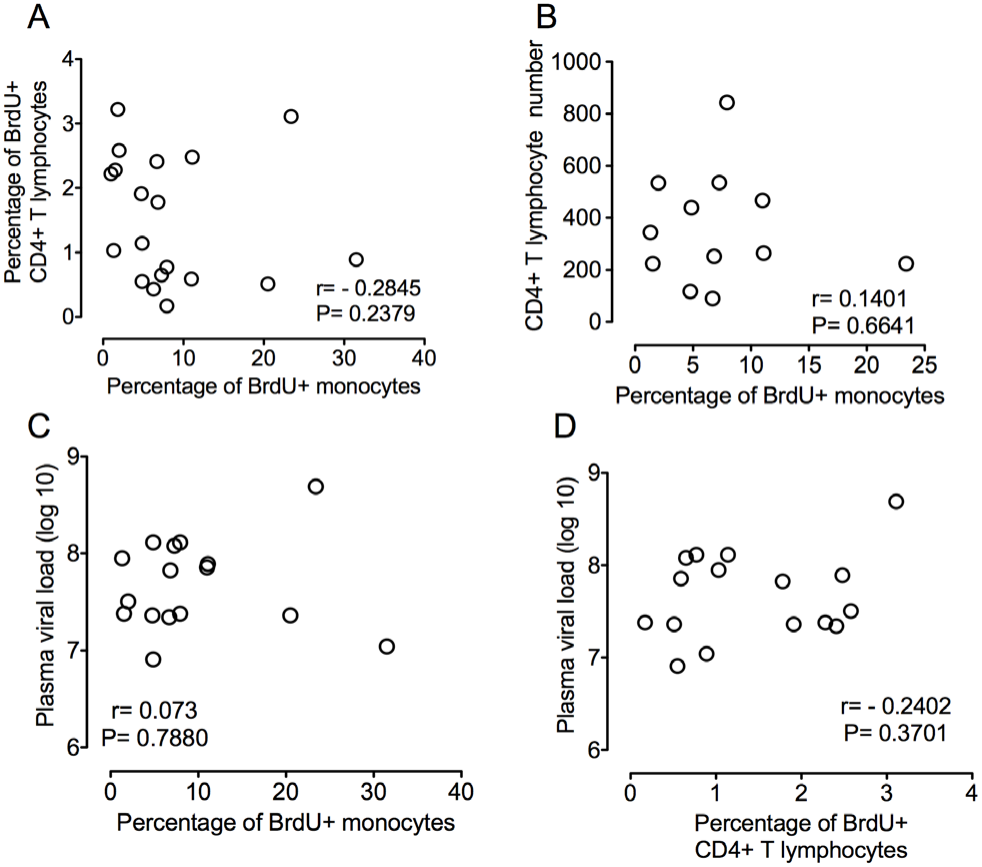
Percentage of BrdU+ monocytes does not correlate with plasma virus or CD4+ T lymphocyte turnover or numbers. **A**. Paired XY values for percentage of BrdU incorporation in CD4+ T lymphocytes and monocytes in all infected animals are plotted. No correlation is found between the percentage of BrdU+ monocytes and the percentage of BrdU+ CD4+ T lymphocytes (P = 0.2379). **B**. Paired XY values for percentage of BrdU incorporation monocytes and CD4+ T lymphocyte counts in all seven infected animals are plotted. No correlation is found between the percentage of BrdU+ monocytes and CD4+ T cell counts (P = 6641). **C**. Paired XY values for percentage of monocyte BrdU incorporation and plasma viral load in all infected animals are plotted. No correlation is found between the percentage of BrdU+ monocytes and plasma viral loads P = 7880. **D**. Paired XY values for percentage of BrdU+ CD4+ T cells and plasma virus in all infected animals are plotted. No correlation is found between the percentage of BrdU+ CD4+ T cells and plasma viral loads P = 0.3701. A Spearman rank test is used for statistics.

To further study if increased monocyte turnover can predict rapid progression to AIDS, we examined other parameters previously linked to disease progression including CD4+ T lymphocyte numbers, CD4+ T lymphocyte turnover, and plasma viral load (Figure 3A–D). Using our model of CD8+ T lymphocyte depletion and SIV infection, plasma viral loads peaked by day 8 and remained high throughout the course of disease, consistent with persistently depleted animals (Table 1) [32], [36]. No correlation was found between percentage of BrdU+ monocytes and percentage of BrdU+CD4+ T lymphocytes (P = 0.2845) or numbers of CD4+ T lymphocytes (P = 0.6641) (Figure 3A and B, respectively). In addition, there was no correlation between plasma virus and the percentage of BrdU+ monocytes (P = 0.7880) or percentage of BrdU+CD4+ T lymphocytes (P = 0.3701) (Figure 3C and D, respectively). Because the number of CD4+ T cells increase with CD8+ lymphocyte depletion, the CD4+ numbers are not comparable to non-CD8 depleted animals [38].

**Figure 4:**
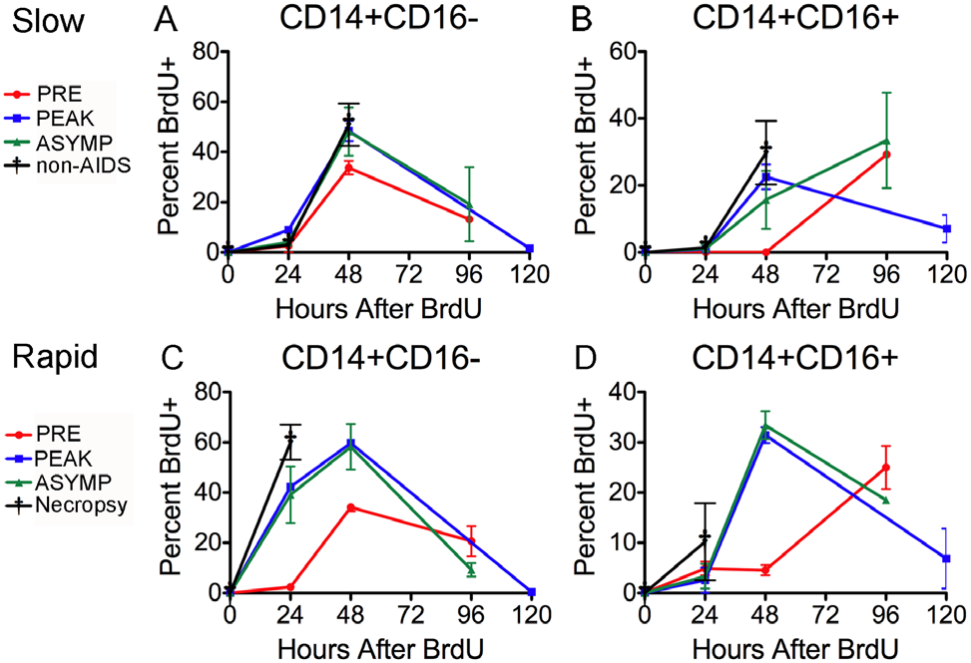
Monocyte subsets leave the bone marrow at different rates that are accelerated in animals that develop SIVE. BrdU was injected four times over the course of the study. Red = pre-infection (days −10 days post infection (dpi)), Blue = peak infection (7 dpi), Green = “asymptomatic” period (26 dpi) and Black = 88 dpi (slow progressors = A–B) or necropsy (rapid progressors = C–D). BrdU injections were given at 24hrs before necropsy. The percentage of BrdU+ monocytes in each subset was determined 24hr and 48hr and either 96 or 120 hrs after BrdU injection (Time: 0 hr). (**A–B**) Dots represent the averages of the percentage of BrdU+ cells in the subsets of slow progressors that remained asymptomatic throughout the period examined. **A**. There is no change in the percentage of BrdU+ CD14+CD16− cells between pre- and post-infection time points. **B**. There is a slight increase in the percentage of BrdU+ CD14+CD16+cells after infection that is apparent at 48hrs. (**C–D**) Dots are averages from two rapid progressors (244-96 and 55-05). The percentage of BrdU+ monocytes was only examined 24hrs after BrdU pulse for animals, DB79 and CM07. **C**. The difference in the percentage of BrdU+CD14+CD16− cells between pre- and post-infection is apparent at 24hrs. **D**. The difference in the percentage of BrdU+ CD14+CD16+cells between pre- and post-infection occurs at 48hrs. The error in all graphs is the standard error of the mean.

We examined the kinetics of BrdU+ monocyte subsets (CD14+CD16− and CD14+CD16+) entering and exiting blood at four time points: pre-infection (9 days before infection), peak infection (7 days post infection), 26 days post infection, and either 88 days post infection (slow progressors) or 24hrs prior to necropsy (rapid progressors) (Figure 4A–B and 4C–D). The percentage of BrdU incorporation was measured at 24hrs and 48hrs and either 96 or 120hrs after a single BrdU injection. First, BrdU incorporation was examined before SIV infection. In all animals, the percentage of “classical” CD14+CD16− monocytes that were BrdU+ peaked in blood 48hrs post BrdU (Figure 4A and C; red lines), whereas the “inflammatory” CD14+CD16+ cells that were BrdU+ peaked at 96hrs post BrdU (Figure 4B and D; red lines). This suggests that CD14+CD16+ cells might arise from CD14+CD16− monocytes in the blood after leaving the bone marrow or that the majority of the CD14+CD16+ cells leave the bone marrow later than the CD14+CD16− cells. Second, the effect of SIV infection on kinetics of BrdU+ monocyte subsets was examined. In contrast to slow progressors, a peak in the percentage of BrdU+CD14+CD16− monocytes in rapid progressors appeared at 24hrs post BrdU after infection (Figure 4C; blue, green, black lines). The percentage of BrdU+CD14+CD16+ monocytes was increased after infection at 48hrs post BrdU in both slow and rapid progressors (Figures 4B and D; blue, green, black lines), with a greater increase in the rapid progressors (Figure 4D). There was no change in any of the monocyte subsets in the uninfected CD8+ T lymphocyte depleted animals (data not shown).

Consistent with previous literature describing an increase of CD14+CD16+ cell numbers in animals with SIVE [25]–[27], [30], [50], [51], the absolute number of CD14+CD16+ monocytes was elevated in the rapid progressors (data not shown). There was no change in the absolute number of CD14+CD16+ monocytes in the slow progressors throughout infection (data not shown).

**Figure 5:**
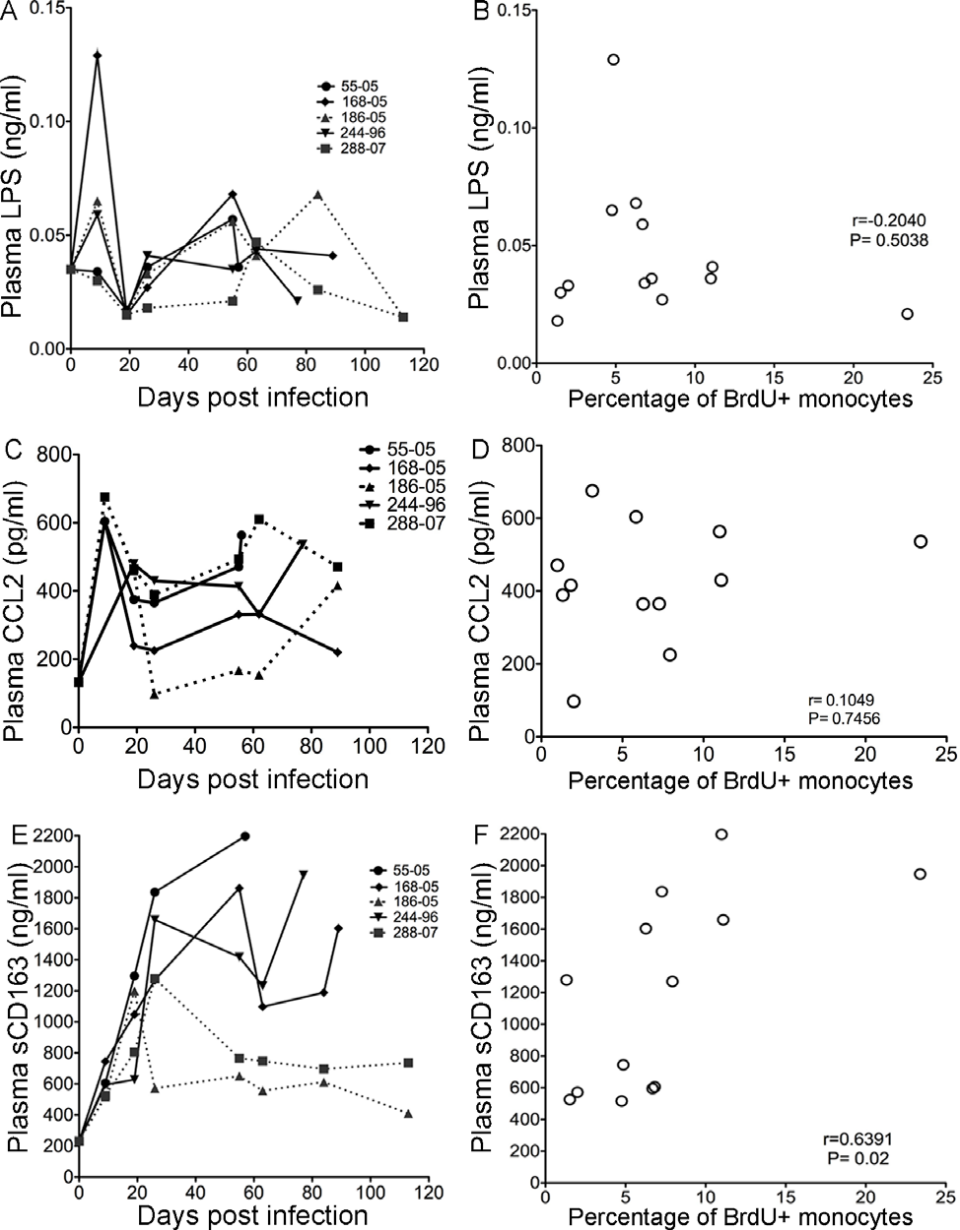
sCD163 levels in plasma, but not LPS or CCL2, correlates with percentage of BrdU+ in total monocytes. **A**. LPS plasma levels in the individual SIV infected animals after infection are shown. Solid lines are plasma LPS levels in rapid progressors. Dashed lines are plasma LPS levels in slow progressors. **B**. There is no correlation between plasma LPS levels with the percentage of BrdU+ monocytes. **C**. CCL2/MCP-1 plasma levels in individual SIV infected animals after infection are shown. The solid lines represent plasma CCL2/MCP-1 levels in rapid progressors. The dashed lines represent plasma CCL2/MCP-1 levels in slow progressors. **D**. There is no correlation between plasma CCL2/MCP-1 and the percentage of BrdU+ monocytes. **E**. sCD163 plasma levels in individual SIV infected animals after infection are shown. Solid lines are sCD163 plasma in rapid progressors. Dashed lines are sCD163 plasma in slow progressors. **F**. There is a significant correlation between sCD163 plasma levels and percentage of BrdU+ monocytes. The Spearman rank test is used.

Recent studies have shown an association of high plasma LPS with increased sCD14 thus implicating monocyte activation in HIV infection [52], [53]. Exact correlates between HIV dementia and plasma LPS were not found [53]. We therefore examined plasma LPS as a potential stimulus for emigration of monocytes from the bone marrow. There were no significant differences in plasma LPS levels between rapid and slow progressors (Figure 5A; rapid progressors = solid lines, slow progressors = dotted lines) and no significant correlation between the percentage of BrdU+ monocytes and plasma LPS levels (Figure 5B; r = −0.2040, P = 0.5038).

To examine whether increased circulating CCL2/monocyte chemoattractant protein 1 (MCP-1) can result in enhanced monocyte emigration from bone marrow, plasma CCL2 levels in SIV-infected animals were examined (Figure 5C). Plasma CCL2 levels increased at 9 days post infection and then moderately decreased in all animals, except one slow progressor whose CCL2 concentration returned to pre-infection levels (Figure 5C; rapid progressors = solid lines, slow progressors = dotted lines). There was no correlation between the percentages of BrdU+ monocytes and plasma CCL2 (Figure 5D; r = 0.1049, P = 0.7456). Interestingly, sCD163 did correlate with BrdU incorporation, consistent with activation of monocyte/macrophages and increased monocyte traffic from the bone marrow (Figure 5E and F) [35], [39]–[42], [45], [54], [55]. In all animals examined, plasma sCD163 levels increased by 9 days post infection, the time point when slow and rapid progressors can be distinguished by differences in the percentage of BrdU+ monocytes (Figure 5E). At day 20, in slow progressors sCD163 plasma levels decreased (Figure 5E, dotted lines), but in rapid progressors sCD163 levels continued to increase (Figure 5E, solid lines). There was a significant correlation between the percentage of BrdU+ monocytes and plasma sCD163 levels (Figure 5F; r = 0.6391, P = 0.02). Thus, sCD163 levels, but not plasma LPS or CCL2, correlated with the increased percentage of BrdU+ monocytes in the blood of CD8+ T lymphocyte depleted SIV-infected macaques.

**Figure 6:**
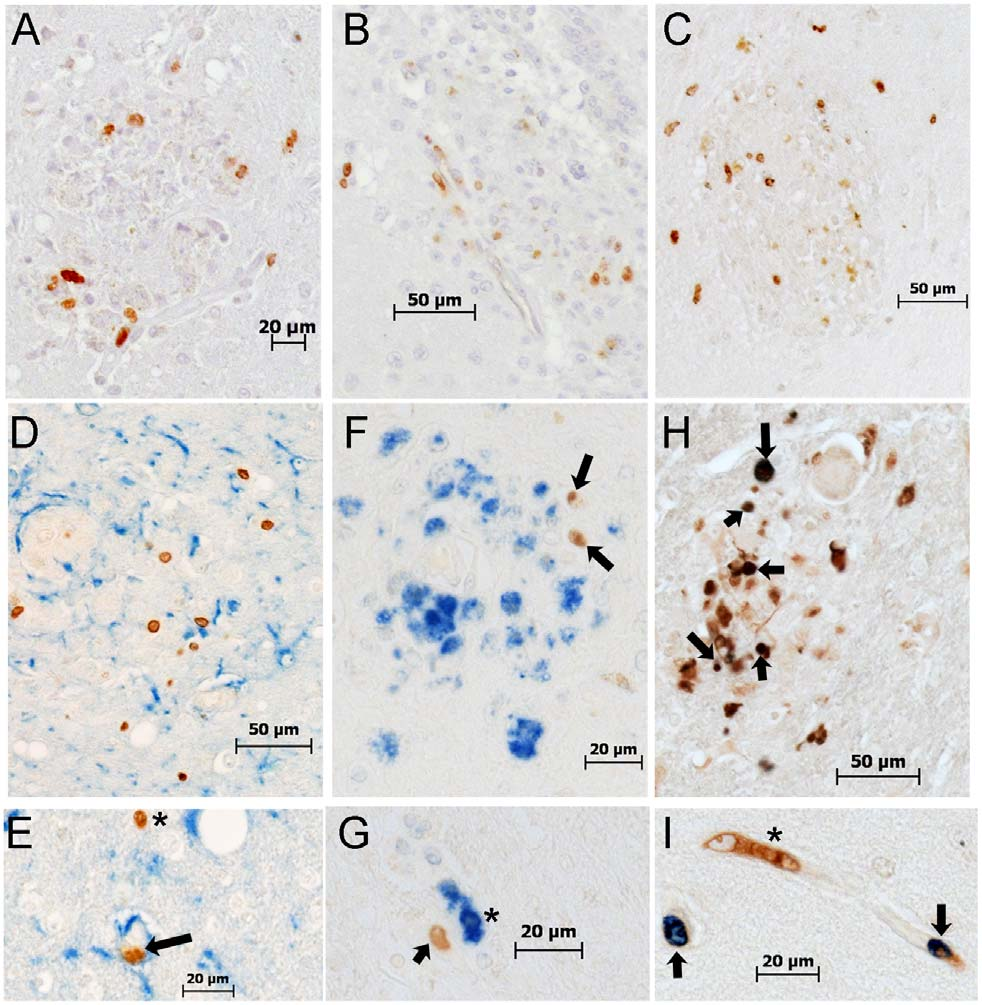
Histopathologic studies showed evidence of BrdU+ cells, the majority of which are Mac387+, in the brains of macaques with SIVE. **A–B**: Immunohistochemistry with an antibody against BrdU is utilized in brain sections of rapid progressors. **A**: BrdU+ cells are present in and around SIVE lesions (BrdU: DAB, brown) **B**: BrdU+ cells are seen in and around the vasculature (BrdU: DAB, brown) **C**: Immunohistochemistry with antibodies against CD3 (Vector Blue, blue) and BrdU (DAB, brown) is utilized in brain sections of rapid progressors to examine if BrdU+ cells are CD4+ T lymphocytes. No double label BrdU+ and CD3+ cells are found in the brains of SIVE+ macaques. **D and E**: Immunohistochemistry with antibodies against GFAP (Vector Blue, blue) and BrdU (DAB, brown) is utilized in brain sections of rapid progressors to examine if BrdU+ cells are astrocytes. There are very few BrdU+ astrocytes seen in all sections examined. The arrow points to a BrdU+ in a vessel surrounded by astrocyte foot processes. The asterisk points out a BrdU+ cells in close proximity to GFAP+ astrocytes. **F and G**: Immunohistochemistry with antibodies against CD68 (DAB, brown) and BrdU (Vector Blue, blue) is utilized in brain sections of rapid progressors to examine if BrdU+ cells were CD68+ mature macrophages. Few BrdU+CD68+ cells are seen. **H and I**: Double staining with antibodies against BrdU (Vector Blue, blue) and Mac387 (DAB, brown) is utilized to determine if BrdU+ cells in the brain are early monocyte/macrophage infiltrates. BrdU and Mac387 did co-localization in the brain of SIVE animals. The arrows indicate BrdU+Mac387+ monocyte/macrophages, in a lesion (**H**) or perivascular region (**I**). The asterisk is a Mac387 BrdU- cell in the vasculature. Brain sections are representative of three SIVE+ animals examined for all stains.

In a recent study, we showed that higher levels of BrdU+ monocytes in blood were associated with macrophage apoptosis in lymph nodes [20]. By flow cytometry, we did not find Annexin V+ BrdU+ monocytes suggesting that monocytes were not undergoing apoptosis in blood (data not shown). Herein, we examined BrdU labeled cells in the CNS investigating their distribution in the brains of three animals, two with severe SIVE and one with mild SIVE (Figure 6). The majority of the BrdU+ cells in all brains were located within SIVE lesions and in the vasculature (Figure 6A and B; BrdU: DAB). BrdU+ cells comprised 15.6 and 17.5% of total cells within SIVE lesions in two rapid progressors with severe SIVE (Table 2, Figure 6A). In the animal with mild SIVE, similar percentages of BrdU+ cells were found in lesions, but there were fewer overall lesions. To determine the identity of the BrdU+ cells, double immunohistochemistry was performed using antibodies against CD3 for T cells, GFAP for astrocytes, CD68 for resident macrophages and Mac387 for recently infiltrated monocyte/macrophages [56], [57]. There was little to no CD3+ T lymphocytes found in any sections examined (Figure 6C; BrdU: DAB and CD3: Vector Blue). BrdU+ cells were found in close proximity to GFAP+ astrocytes, but very few scattered double positive astrocytes (GFAP+BrdU+) were found (Figure 6D and E; BrdU: DAB and GFAP: Vector Blue). Approximately 10% of total BrdU+ cells in SIVE lesions were CD68+ and these BrdU+ cells comprised between 1.8 and 4.6% of all CD68+ macrophages in lesions (Figure 6F and G; BrdU: DAB and CD68: Vector Blue and Table 2). Between 81 to 92% of all BrdU+ cells in SIVE lesions were Mac387+, representing approximately a third of the total Mac387+ monocyte/macrophages in lesions (Figure 6H and I; BrdU: Vector Blue and Mac387: DAB and Table 2). The animal with mild SIVE had similar percentages of BrdU+Mac387+ and BrdU+CD68+ cells in lesions as the animals with severe SIVE. In the brains of control non-infected CD8+ T lymphocyte depleted animals, few scattered BrdU+ macrophages were detected, representing normal monocyte traffic (data not shown).

**Table 2:** Percentages of BrdU+, Mac387+, CD68+ and SIVp28+ cells in SIVE lesions.

	Animal 244-96	Animal DB79
BrdU+ cells vs. all cells#	15.6±1.5; n = 34	17.5±1.7; n = 19
BrdU+Mac387+ cells vs. all BrdU+ cells*	81.0±7.8; n = 14	92.2±4.7; n = 11
BrdU+CD68+ cells vs. all BrdU+ cells**	9.8±3.0; n = 31	10.7±7.6; n = 5
BrdU+SIVp28+ cells vs. all BrdU+ cells***	0.78±0.39; n = 27	0.48±0.48; n = 14
BrdU+Mac387+ cells vs. all Mac387+ cells‡	34.0±4.9; n = 14	45.3±8.3; n = 11
BrdU+CD68+ cells vs. all CD68+ cells‡‡	1.8±0.5; n = 31	4.6±3.9; n = 5
BrdU+SIVp28+ cells vs. all SIVp28+ cells‡‡‡	0.12±0.062; n = 27	0.19±0.19; n = 14

# Mean±SEM (standard error of the mean) of the percentage of BrdU+ cells in SIVE lesions calculated as followed; (number of BrdU+ cells/total number of cells in lesions using hematoxylin) ×100.

*Mean±SEM of the percentage of BrdU+ cells expressing Mac387 in SIVE lesions calculated as followed; (number of BrdU+Mac387+ cells/total number of BrdU+ cells) ×100.

**Mean±SEM of the percentage of BrdU+ cells expressing CD68 in SIVE lesions calculated as followed; (number of BrdU+CD68+ cells/total number of BrdU+ cells) ×100.

***Mean±SEM of the percentage of BrdU+ cells expressing SIVp28 in SIVE lesions calculated as followed; (number of BrdU+SIVp28+ cells/total number of BrdU+ cells) ×100.

**‡:** Mean±SEM of the percentage of Mac387+ cells expressing BrdU in SIVE lesions calculated as followed; (number of BrdU+Mac387+ cells/total number of Mac387+ cells) ×100.

**‡‡:** Mean±SEM of the percentage of CD68+ cells expressing BrdU in SIVE lesions calculated as followed; (number of BrdU+CD68+ cells/total number of CD68+ cells) ×100.

**‡‡‡:** Mean±SEM of the percentage of SIVp28+ cells expressing BrdU in SIVE lesions calculated as followed; (number of BrdU+SIVp28+ cells/total number of SIVp28+ cells) ×100.

n = number of total lesions examined. For 244-96 frontal cortex, prefrontal cortex and basal nuclei and for DB79 frontal cortex and occipital cortex sections were examined. The mild SIVE animal, 55-05, had similar percentages of BrdU+ cells, BrdU+Mac387+ and BrdU+CD68+ cells in lesions compared to DB79 and 244-96, but there were fewer lesions overall.

**Figure 7:**
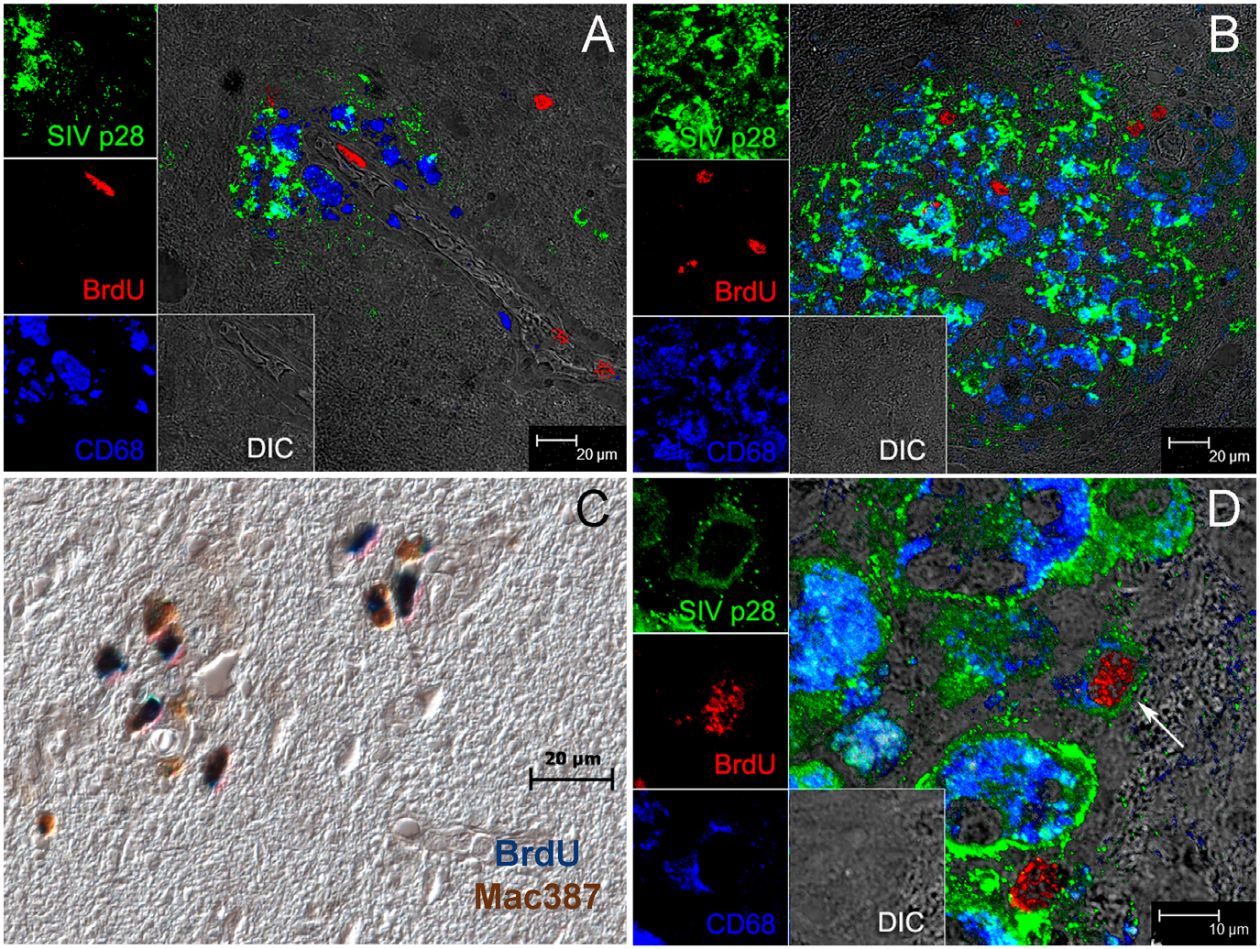
The majority of BrdU+ monocyte/macrophages in the brain are not productively infected. Triple label with antibodies against BrdU (red), SIV p28 (green), CD68 (blue) is used to determine if BrdU+ cells are productively infected. Side panels are single-color images of SIVp28 (green), BrdU (red), CD68 (blue) and differential interference contrast (DIC). **A**: BrdU+ (red) cells in blood vessels and a BrdU+ cell infiltrated in the brain **B–D**: Multiple BrdU+ cells are detected in and around SIVE lesion. **B**: BrdU+ cells are seen in and around this SIVE lesion; the majority BrdU+ cells are SIV p28− and thus not productively infected. **C**: The majority of the BrdU+ cells in lesions are Mac387+. Double label with antibodies against BrdU (blue) and Mac387 (brown) in an SIVE lesion is shown. **D**: An SIVE lesion in the brain of a rapid progressor showing a rare triple positive cell: SIV-infected CD68+BrdU+ macrophage (white arrow). Data presented here are representative of *n* = 3 macaques with SIVE. Multiple tissue sections from different brain regions were examined.

Less than 1% of all BrdU+ cells in SIVE lesions were SIVp28+ and less than 0.2% of all SIVp28+ cells were also BrdU+ (Table 2). Double positive BrdU and SIVp28 cells are very rare events, thus, resulting in few productively infected BrdU monocyte/macrophages. Immunofluorescence confirmed the presence of numerous BrdU+ cells in blood vessels (Figure 7A), perivascular cuffs (Figure 7A), and SIVE lesions (Figure 7B–D) and showed that the majority of the BrdU+ cells were Mac387+ cells (Figure 7C; BrdU: Vector Blue and Mac387: DAB) that were not productively infected. Immunofluorescence was used to identify that the productively SIV infected BrdU+ cells were CD68+ macrophages (Figure 7A–B and 7D; white arrow). Thus, the majority of the BrdU+ cells in the brain were Mac387+ that were not productively infected, representing monocyte/macrophages that were labeled with BrdU in the bone marrow and had recently trafficked to the brain (likely from the last two BrdU pulses). These data are consistent with Mac387 as one of the earliest differentiation markers expressed on monocyte/macrophages as they enter tissues [56], [57].

## Discussion

Here we have presented data showing that increased monocyte turnover is predictive of rapid development of AIDS. We have demonstrated that an increased percentage of BrdU+ monocytes in blood correlated with the severity of SIVE at necropsy. Interestingly, the differences in the percentage of BrdU+ monocytes were apparent by 8 days post infection and differentiated between slow and rapid progression by 27 days post infection. This data lends support to the importance of early monocyte activation and deregulation in AIDS pathogenesis. Furthermore, sCD163 levels, but not plasma LPS or CCL2, correlated with increased percentages of BrdU+ monocytes in the blood of SIV-infected macaques. In addition, we showed a differential rate of turnover in two major monocyte populations (CD14+CD16− and CD14+CD16+) and the acceleration of their turnover in rapid progressors. BrdU+ cells were detected in the brains of animals with SIVE; the majority of these cells were Mac387+ that were SIV p28− and a minor population of CD68+ macrophages, few of which were productively infected. The data presented here underscore the importance of increased monocyte turnover and traffic to the brain during SIVE, and emphasize in this model that CNS lesion formation is an active process requiring monocyte/macrophage recruitment, likely a result of enhanced innate immune responses [27], [35].

These data confirm and extend the observations recently reported by Hasegawa and colleagues, who examined BrdU incorporation in animals at different stages of SIV infection and found an increase in the percentage of BrdU+ monocytes with acute and chronic infection and a greater expansion with AIDS [20]. Our results are consistent with that finding, but add the observation that increased BrdU incorporation in monocytes correlates with the severity of SIVE and can distinguish between slow and rapid progressors.

We have presented evidence that increased monocyte traffic from bone marrow into the circulation correlates with the rapid progression to AIDS and severity of SIVE. BrdU+ monocytes traffic from bone marrow through the circulation into the brain, fostering the development of AIDS and SIVE. An increase in monocyte traffic in uninfected animals or slow progressors was not found. These data underscore the important role of monocyte activation and augmented traffic from the bone marrow to the brain in SIV neuropathogenesis. Bone marrow diffusion has been reported to correlate with the incidence of HIV dementia [58]. Additionally, anemia before the onset of AIDS is predictive of HIV neuropathogenesis [35], [58]. It has been demonstrated in SIV infected monkeys that rapid disease progression is the best correlate with the development of SIVE [59]. Our data support these observations and extend these to include that the rate of monocyte turnover, demonstrated by the percentage of BrdU+ monocytes, correlates with the rapid development of AIDS and the severity of SIVE.

The CD8 depletion, SIV infection model produces rapid AIDS with a high incidence of SIVE by depleting cytotoxic T lymphocytes. Using this model, there were both slow and rapid disease progressing animals with varying degrees of severity of SIVE that correlated with the percentage of BrdU+ monocytes. However, CD4+ T lymphocytes, which are linked to CD8+ T lymphocytes, are not regulated in a normal fashion due to depletion of CD8+ T lymphocytes [38], a possible reason for the lack of a correlation between CD4+ T lymphocytes with BrdU+ monocytes in our study. However, in a previous report using non-CD8 depleted SIV infected monkeys, high monocyte turnover also did not correlate with CD4+T cell number or plasma viral load [20].

We found that the percentage of BrdU+ monocytes does not correlate with LPS or CCL2 levels in plasma, but interestingly sCD163 did; this is consistent with the notion that activation of monocyte/macrophages and the increase of monocyte traffic from the bone marrow drive CNS pathogenesis [39], [40]. Previous in vivo and in vitro studies have shown that CD163 expression on monocytes inversely correlated with sCD163 in plasma or tissue culture media directly linking sCD163 to monocyte activation [39], [45], [55]. In vitro studies show picogram levels of LPS results in sCD163 release from monocytes, underscoring the role of innate immune responses in AIDS pathogenesis [45]. In addition, the level of sCD163 has been shown to increase in association with macrophage mediated diseases, including sepsis [40] and Gaucher's disease [60], characterized by macrophage accumulation in the liver and spleen [60]. Thus, increased traffic of monocytes from the bone marrow (increased BrdU+ monocytes in the circulation) and increased levels of sCD163 in the plasma are consistent with an inflammatory environment resulting from stimulation of innate immunity that may play important roles in the development of SIVE.

Release of monocytes from the bone marrow could be triggered by increased apoptosis of monocytes in the blood. Although CD3+CD4+ T cells from HIV-1 subjects have been shown to undergo constitutive and induced apoptosis [61], HIV-1+ monocytes are resistant [61]. Recently, it was demonstrated that peripheral blood monocytes from chronically HIV-1 infected individuals have a stable anti-apoptotic gene signature suggesting a greater resistance to apoptosis in circulating monocytes during HIV infection [61]. In this study, pro-apoptotic genes were down-regulated and anti-apoptotic genes were up-regulated in infected monocytes [61]. When we examined whole blood by flow cytometry for the presence of apoptotic monocytes, we did not detect monocytes that were Annexin V and propidium iodide (PI) positive 24hrs after BrdU injection (data not shown). Thus, we could rule out apoptosis of peripheral blood monocytes as a trigger for the increased release of monocytes from the bone marrow. Apoptosis of tissue macrophages in lymph nodes has already been demonstrated to correlate with the percentage of BrdU monocyte label in blood [20].

A report by Serbina and Pamer suggested that monocyte emigration from bone marrow during bacterial infection requires signals mediated by the chemokine receptor CCR2 [62]. Using a *Ccr2−/−* mouse model, it was found that CCR2 is important for release of Ly6C^hi^ monocytes (equivalent to the CD14+CD16− monocytes in humans) from bone marrow [62]. A more recent paper concluded that not only CCL2, but also CCL7 (MCP-3) was critical in monocyte mobilization from the bone marrow [63]. We did not see a correlation between CCL2 plasma levels and the percentage of BrdU+ monocytes, but that does not rule out the involvement of CCR2 and additional ligands. In addition, the chemokine CXCL12 (SDF-1) and its receptor CXCR4 are known to be involved in the retention of hematopoietic stem cells in the bone marrow [64], [65]. The roles of these additional factors are worthy of exploration in the SIV model of disease and may lead to further insight into the increased release of monocytes from the bone marrow during AIDS and SIVE progression.

Not only did we find differences in the percent of BrdU+ total monocytes between slow and rapid progressors, but we found kinetic differences in BrdU incorporation of two monocyte subsets, CD14+CD16− and CD14+CD16+ entering the blood from the bone marrow. In the rapid progressors, the percentage of BrdU incorporation was increased dramatically with infection in both populations. The CD14+CD16− (CCR2+) monocyte population may have accelerated release due to increased levels of MCP-3, as discussed above. The CD14+CD16+ monocyte blood population has been shown to be phenotypically similar to perivascular macrophages in the brain, which supports the concept that the CD14+CD16+ monocytes transmigrate into the brain to differentiate further into perivascular macrophages [25], [33]. Our data suggests that although CD14+CD16+ cells leave the bone marrow later than CD14+CD16− cells, the CD14+CD16+ cells spend less time in the circulation, possibly a result of traffic to tissues. This likelihood is supported by the finding that CD14+CD16+ monocytes are inflammatory mediators expressing high levels of pro-inflammatory cytokines and are potent antigen presenting cells. We have not ruled out the possibility that CD14+CD16− monocytes convert to CD14+CD16+ monocytes in blood [66].

The overall number of brain macrophages in HIVE and SIVE is increased despite evidence of proliferation in situ, supported by the notion that the majority of these macrophages are derived from the periphery [25], [34]. Here, we present evidence that newly infiltrated BrdU+ monocyte/macrophages are a significant population of monocyte/macrophages in perivascular cuffs and lesions in brains of SIVE+ animals. Recruited monocytes may increase the number of viral target cells in the brain. Most of the BrdU+ cells were Mac387+ cells representing newly recruited and infiltrated monocytes/macrophages [56], [57]. It is not known how long the BrdU+Mac387+ cells stay in the CNS with lesion formation nor at which BrdU pulse these cells were labeled in the periphery. This might, in part, explain why CD68+BrdU+ cells are found in the CNS; they might have trafficked earlier as less mature cells and undergone maturation within tissues. Additionally, the CD68+ BrdU+ cells could have migrated directly from the blood, thus bringing HIV into the CNS. Lastly, they may represent macrophages that divided in the CNS, although we believe this in unlikely since we found little evidence of BrdU+ microglia, astrocytes or epithelial cells in our studies. We have previously shown using specific markers for cell proliferation, including Ki-67 and topoisomerase II alpha, that macrophages within SIVE lesions were not undergoing significant active proliferation in the time frame studied [34].

Increased monocyte turnover correlated with high levels of sCD163 in plasma. These findings along with potential anti-inflammatory properties of sCD163 [44], which is directly shed from the M2 anti-inflammatory alternative activated macrophages [39], [67], suggest that BrdU+ cells recruited to the CNS may aid in lesion resolution. Monocyte/macrophages may traffic to the brain as inflammatory cells in response to or causing neuronal injury or as anti-inflammatory vehicles to minimize injury to the brain during SIV infection.

Although we detected very few SIVp28+ BrdU+ cells in SIVE brains, this does not rule out the possibility that the BrdU+ cells are not latently infected. In fact, our data suggests BrdU+ cells may act as viral reservoirs that may with maturation actively replicate virus. Our finding that only the BrdU+CD68+ monocytes are productively infected underscores the importance of monocyte/macrophage maturation (possibly Mac387 to CD68 expression) for active viral replication. Invasion of the CNS by BrdU+Mac387+ perivascular macrophages with concomitant viral infection of the CNS compartment upon maturation is a likely scenario for this. Overall, our data examining BrdU+ monocytes in blood and BrdU+ monocyte/macrophages with the brain, underscore the role of monocyte/macrophages derived from bone marrow in AIDS pathogenesis and CNS disease.

## Materials and Methods

### Ethics statement

All animals were handled in strict accordance with good animal practice as defined by the Harvard University's Institutional Animal Care and Use Committee, and all animal work was approved by this committee.

### Animals, viral infection and CD8+ T lymphocyte depletion

Eleven rhesus macaques were utilized in this study. Seven animals were infected with SIVmac251 (20 ng of SIV p27) by intravenous injection, kindly provided by Ronald Desrosiers. In order to achieve rapid disease progression with high incidence of SIVE, animals were CD8+ T lymphocyte depleted by treatment with a human anti-CD8 antibody cM-T807 administered s.c. (10mg/kg) at 6 days post infection and i.v. (5mg/kg) at 8 and 12 days post infection (previously described [32]). Four uninfected animals were used as controls: two were CD8+ T lymphocyte depleted by treatment with a human anti-CD8 antibody cM-T807 administered once (50mg/kg) i.v. and two were CD8+ T lymphocyte depleted by treatment with a rhesus anti-CD8 antibody cM-T807 administered once (50mg/kg) i.v. These antibodies were provided by the NIH Non-human Primate Reagent Resource (RR016001, AI040101). CD8+ T lymphocyte depletion was monitored by flow cytometry prior to antibody treatment and weekly thereafter during infection (as previously described [32]). All animals were housed at Harvard University's New England Regional Primate Research Center in accordance with standards of the American Association for Accreditation of Laboratory Animal Care. Animals were anesthetized with ketamine-HCl and euthanized by an intravenous pentobarbital overdose and exsanguinated. Animals were sacrificed with any of the following 5 criteria: 1. weight loss >15% body weight in 2 weeks or >30% body weight in 2 months, 2. documented opportunistic infection, 3. persistent anorexia >3 days without explicable cause, 4. severe intractable diarrhea, progressive neurological signs or 5. significant cardiac and/or pulmonary signs. The diagnosis of AIDS was determined by the presence of AIDS defining lesions: Pneumocystis pneumonia, Mycobacterium avium infection (most commonly small intestine, liver and mesenteric lymph node), intestinal adenovirus infection (most common in small intestine). Other, less common lesions include SIV giant cell disease in the lung, gut, and lymph nodes and SIV associated arteriopathy. SIV encephalitis (SIVE) was defined by the presence of multinucleated giant cells (MNGC) and accumulation of macrophages, some of which are infected [59], [68]–[70]. No animals with opportunistic infections in the CNS were used in this study.

### Viral load determination

Plasma SIV RNA was quantified using real-time PCR as previously described [71]. SIV virions were pelleted from 0.5ml EDTA plasma by centrifugation at 20,000 g for 1 hour. The fluorescently labeled, real-time PCR probe employed contained a non-fluorescent quencher, BHQ-1, at its 3′ end. The threshold sensitivity was 100 copy Eq/ml, with an average interassay coefficient of variation of less than 25%.

### BrdU administration

A 30 mg/ml stock of solution was prepared by adding 5-bromo-2′-deoxyuridine (BrdU) (Sigma) to 1× PBS (without Ca^2+^ and Mg^2+^), U.S.P. grade (Aestus Pharmaceuticals) and heated to 60°C in water bath (as previously described [20], [24]). BrdU was administered as a slow bolus i.v. injection at a dose of 60 mg BrdU/kg body weight. In the uninfected animals, BrdU was given four times throughout the study (days −11, 3, 38 and 72 days post-CD8 antibody administration). In the infected animals, BrdU was administered pre-infection (day −9), peak infection (day 7), and days 26, day 50 (n = 1) and 88 (n = 2) post infection and 24 hours prior to necropsy (n = 4; days 55, 76, 88 and 91). CM07 did not receive BrdU prior to necropsy.

### Flow cytometry

Flow cytometric analyses were performed with 100 µl aliquots of EDTA-coagulated whole blood. Erythrocytes were lysed using ImmunoPrep Reagent System (Beckman Coulter), washed twice with PBS containing 2% FBS, then incubated for 15 minutes at room temperature with fluorochrome-conjugated surface antibodies including anti-HLA-DR-PerCp-Cy5.5 (clone L243), anti-CD16-PE-Cy7 (clone 3G8; BD Biosciences), anti-CD3-APC (clone SP34-2), anti-CD8-APC (clone RPA-T8;), anti-CD20-APC (clone 2H7) and anti-CD14-Pacific blue (clone M5E2) (BD Biosciences). For intracellular staining, cells were fixed and permeabilized with BD Cytofix/Cytoperm™ buffer (BD Biosciences) for 30 mins at room temperature. Cells were again washed and incubated with BD Cytoperm Plus™ buffer for 10 mins on ice, then washed and incubated with DNase (30mg) for 1hr at 37°C, washed and then stained for intracellular antigen with anti-BrdU-FITC (clone 3D4; BD Biosciences) and anti-Ki-67-PE (clone B56; BD Biosciences) for 20 mins at room temperature. For controls, BrdU naïve animals and isotype controls were used. To test for apoptotic monocytes anti-Annexin V (Invitrogen) and propidium iodine (PI; BD Biosciences) were used. Samples were acquired on a BD FACS Aria (BD Biosciences) and analyzed with Tree Star Flow Jo version 8.7.

### sCD163 and CCL2 ELISAs and LAL assay

Soluble CD163 (sCD163) and CCL2 plasma levels were quantified by ELISA according to manufacturer's protocol (Trillium Diagnostics and R&D Systems, respectively). The Diazo-coupled Limulus amebocyte lysate (LAL) assay (Associates of Cape Cod Inc.) was used to quantify endotoxin/liposaccharide (LPS) levels in plasma from SIV-infected animals, according to the manufacturer's protocol. Briefly, samples diluted 1/5 were inactivated for 30 min at 65°C and incubated with LAL for 30 min at 37°C. Addition of reagents led to formation of a magenta derivative that absorbs light at 570 nm. For LAL assay, samples were handled with non-pyrogenic plastic or glassware to avoid LPS contamination.

### Immunohistochemistry

Formalin-fixed, paraffin-embedded brain tissues were deparaffinized and assessed by immunohistochemistry for BrdU (Mouse IgG1; Dako, 1∶50, 1hr room temperature. Before primary antibody incubation, non-serum protein block was applied. The EnVision+ System- horseradish peroxidase (HRP) (EnVision+ Kit; DAKO) was used according to the manufacturers' instructions. The color reaction product was developed using 3,3′-diaminobenzidine tetrahydrochloride (DAB; DAKO) as the chromogenic substrate for HRP. The sections were counterstained with hematoxylin and then dehydrated and mounted. Controls consisted of the addition of isotype-matched immunoglobulin. To detect BrdU^+^ cells and CD3+ T lymphocytes, GFAP+ astrocytes, SIVp28+ infected cells, CD68+ macrophages and Mac387+ monocyte/macrophages in monkey brains, double-label immunohistochemistry was performed using the DAKO Double Stain System, according to the manufacturer's instructions. The color reaction product was developed using DAB and Vector Blue (Vector Laboratories). Sections were visualized under a Zeiss Axio Imager M1 microscope (Carl Zeiss MicroImaging, Inc., Thornwood, NY) using Plan-Apochromat ×20/0.8 and ×40/0.95 Korr objectives.

### Confocal microscopy

Tissues were collected in 10% neutral buffered formalin, and embedded in paraffin. Tissues were sectioned at 6 µm and deparaffinized with xylene and hydrated in graded alcohols. Immunohistochemical staining followed a basic protocol using a citrate antigen retrieval method. For immunofluorescence, sections were blocked with 10% normal goat serum (NGS) in PBS with 0.2% Fish Skin Gelatin (FSG) (Sigma) for 40 min. Tissues were incubated with rat anti-BrdU (IgG2a; BU1/75, Novus Biologicals, 1∶50, 1hr RT) followed by AlexaFluor 568 conjugated goat anti-rat IgG (Molecular Probes; 1∶500), then with mouse anti-SIV p28 (IgG1; Microbix Biosystems, 1∶500) followed by AlexaFluor 488 conjugated goat anti-mouse IgG1 (Molecular Probes; 1∶500) and then CD68-biotin conjugated (1∶20, overnight at 4°C) followed by streptavidin conjugated AlexaFluor 568 (Molecular Probes; 1∶500). After immunofluorescence labeling and washing, sections were treated with 50 mmol/L CuSO_4_ ammonium buffer for 45 minutes to quench auto-fluorescence. Confocal microscopy was performed using a Leica TCS SP2 laser-scanning microscope equipped with 3 lasers (Leica Microsystems, Exton, PA). Individual optical slices represent 0.2 µm. Optical slices were collected at 512×512 pixel resolution. The fluorescence of individual fluorochromes was captured separately in a sequential mode, after optimization to reduce bleed-through between channels (photomultiplier tubes) using Leica software. NIH Image v1.62 and Adobe Photoshop v7 software were used to assign correct colors of up to four channels collected (3 fluorochromes: Alexa 488 (green), Cy3, Alexa 568 (red), and Alexa 647 (far red), and the differential interference contrast image (gray scale).

### Statistical analysis

For statistical analyses we used the Prism version 5.0a (GraphPad Software, Inc., San Diego, CA) software. Spearman rank test was used for all correlations.

## References

[1] Soulas C, Donahue RE, Dunbar CE, Persons DA, Alvarez X (2009). Genetically modified CD34+ hematopoietic stem cells contribute to turnover of brain perivascular macrophages in long-term repopulated primates.. Am J Pathol.

[2] Gonzalez-Mejia ME, Doseff AI (2009). Regulation of monocytes and macrophages cell fate.. Front Biosci.

[3] van Furth R (1989). Origin and turnover of monocytes and macrophages.. Curr Top Pathol.

[4] Tushinski RJ, Oliver IT, Guilbert LJ, Tynan PW, Warner JR (1982). Survival of mononuclear phagocytes depends on a lineage-specific growth factor that the differentiated cells selectively destroy.. Cell.

[5] Landsman L, Varol C, Jung S (2007). Distinct differentiation potential of blood monocyte subsets in the lung.. J Immunol.

[6] Whitelaw DM (1972). Observations on human monocyte kinetics after pulse labeling.. Cell Tissue Kinet.

[7] Varol C, Yona S, Jung S (2009). Origins and tissue-context-dependent fates of blood monocytes.. Immunol Cell Biol.

[8] Sawyer RT, Strausbauch PH, Volkman A (1982). Resident macrophage proliferation in mice depleted of blood monocytes by strontium-89.. Lab Invest.

[9] Tarling JD, Lin HS, Hsu S (1987). Self-renewal of pulmonary alveolar macrophages: evidence from radiation chimera studies.. J Leukoc Biol.

[10] Wijffels JF, de Rover Z, Beelen RH, Kraal G, van Rooijen N (1994). Macrophage subpopulations in the mouse spleen renewed by local proliferation.. Immunobiology.

[11] Swirski FK, Nahrendorf M, Etzrodt M, Wildgruber M, Cortez-Retamozo V (2009). Identification of splenic reservoir monocytes and their deployment to inflammatory sites.. Science.

[12] van Furth R, Diesselhoff-den Dulk MM (1984). Dual origin of mouse spleen macrophages.. J Exp Med.

[13] Nahrendorf M, Swirski FK, Aikawa E, Stangenberg L, Wurdinger T (2007). The healing myocardium sequentially mobilizes two monocyte subsets with divergent and complementary functions.. J Exp Med.

[14] Arnold L, Henry A, Poron F, Baba-Amer Y, van Rooijen N (2007). Inflammatory monocytes recruited after skeletal muscle injury switch into antiinflammatory macrophages to support myogenesis.. J Exp Med.

[15] Van Furth R, Diesselhoff-den Dulk MC, Mattie H (1973). Quantitative study on the production and kinetics of mononuclear phagocytes during an acute inflammatory reaction.. J Exp Med.

[16] Volkman A, Collins FM (1974). The cytokinetics of monocytosis in acute salmonella infection in the rat.. J Exp Med.

[17] Ohgami M, Doerschuk CM, Gie RP, English D, Hogg JC (1991). Monocyte kinetics in rabbits.. J Appl Physiol.

[18] Meuret G, Bammert J, Hoffmann G (1974). Kinetics of human monocytopoiesis.. Blood.

[19] Goto Y, Hogg JC, Suwa T, Quinlan KB, van Eeden SF (2003). A novel method to quantify the turnover and release of monocytes from the bone marrow using the thymidine analog 5′-bromo-2′-deoxyuridine.. Am J Physiol Cell Physiol.

[20] Hasegawa A, Liu H, Ling B, Borda JT, Alvarez X (2009). The level of monocyte turnover predicts disease progression in the macaque model of AIDS.. Blood.

[21] Brown KN, Wijewardana V, Liu X, Barratt-Boyes SM (2009). Rapid influx and death of plasmacytoid dendritic cells in lymph nodes mediate depletion in acute simian immunodeficiency virus infection.. PLoS Pathog.

[22] Shih CH, van Eeden SF, Goto Y, Hogg JC (2005). CCL23/myeloid progenitor inhibitory factor-1 inhibits production and release of polymorphonuclear leukocytes and monocytes from the bone marrow.. Exp Hematol.

[23] Goto Y, Hogg JC, Whalen B, Shih CH, Ishii H (2004). Monocyte recruitment into the lungs in pneumococcal pneumonia.. Am J Respir Cell Mol Biol.

[24] Wang X, Das A, Lackner AA, Veazey RS, Pahar B (2008). Intestinal double-positive CD4+CD8+ T cells of neonatal rhesus macaques are proliferating, activated memory cells and primary targets for SIVMAC251 infection.. Blood.

[25] Fischer-Smith T, Croul S, Sverstiuk AE, Capini C, L'Heureux D (2001). CNS invasion by CD14+/CD16+ peripheral blood-derived monocytes in HIV dementia: perivascular accumulation and reservoir of HIV infection.. J Neurovirol.

[26] Kim WK, Avarez X, Williams K (2005). The role of monocytes and perivascular macrophages in HIV and SIV neuropathogenesis: information from non-human primate models.. Neurotox Res.

[27] Kim WK, Corey S, Alvarez X, Williams K (2003). Monocyte/macrophage traffic in HIV and SIV encephalitis.. J Leukoc Biol.

[28] Williams KC, Hickey WF (1995). Traffic of hematogenous cells through the central nervous system.. Curr Top Microbiol Immunol.

[29] Kedzierska K, Crowe SM (2002). The role of monocytes and macrophages in the pathogenesis of HIV-1 infection.. Curr Med Chem.

[30] Pulliam L, Gascon R, Stubblebine M, McGuire D, McGrath MS (1997). Unique monocyte subset in patients with AIDS dementia.. Lancet.

[31] Thieblemont N, Weiss L, Sadeghi HM, Estcourt C, Haeffner-Cavaillon N (1995). CD14lowCD16high: a cytokine-producing monocyte subset which expands during human immunodeficiency virus infection.. Eur J Immunol.

[32] Williams K, Westmoreland S, Greco J, Ratai E, Lentz M (2005). Magnetic resonance spectroscopy reveals that activated monocytes contribute to neuronal injury in SIV neuroAIDS.. J Clin Invest.

[33] Williams KC, Corey S, Westmoreland SV, Pauley D, Knight H (2001). Perivascular macrophages are the primary cell type productively infected by simian immunodeficiency virus in the brains of macaques: implications for the neuropathogenesis of AIDS.. J Exp Med.

[34] Williams K, Schwartz A, Corey S, Orandle M, Kennedy W (2002). Proliferating cellular nuclear antigen expression as a marker of perivascular macrophages in simian immunodeficiency virus encephalitis.. Am J Pathol.

[35] Williams KC, Hickey WF (2002). Central nervous system damage, monocytes and macrophages, and neurological disorders in AIDS.. Annu Rev Neurosci.

[36] Schmitz JE, Simon MA, Kuroda MJ, Lifton MA, Ollert MW (1999). A nonhuman primate model for the selective elimination of CD8+ lymphocytes using a mouse-human chimeric monoclonal antibody.. Am J Pathol.

[37] Schmitz JE, Johnson RP, McClure HM, Manson KH, Wyand MS (2005). Effect of CD8+ lymphocyte depletion on virus containment after simian immunodeficiency virus SIVmac251 challenge of live attenuated SIVmac239delta3-vaccinated rhesus macaques.. J Virol.

[38] Schmitz JE, Kuroda MJ, Santra S, Sasseville VG, Simon MA (1999). Control of viremia in simian immunodeficiency virus infection by CD8+ lymphocytes.. Science.

[39] Davis BH, Zarev PV (2005). Human monocyte CD163 expression inversely correlates with soluble CD163 plasma levels.. Cytometry B Clin Cytom.

[40] Moller HJ, Peterslund NA, Graversen JH, Moestrup SK (2002). Identification of the hemoglobin scavenger receptor/CD163 as a natural soluble protein in plasma.. Blood.

[41] Buechler C, Ritter M, Orso E, Langmann T, Klucken J (2000). Regulation of scavenger receptor CD163 expression in human monocytes and macrophages by pro- and antiinflammatory stimuli.. J Leukoc Biol.

[42] Fabriek BO, Dijkstra CD, van den Berg TK (2005). The macrophage scavenger receptor CD163.. Immunobiology.

[43] Kim WK, Alvarez X, Fisher J, Bronfin B, Westmoreland S (2006). CD163 identifies perivascular macrophages in normal and viral encephalitic brains and potential precursors to perivascular macrophages in blood.. Am J Pathol.

[44] Moestrup SK, Moller HJ (2004). CD163: a regulated hemoglobin scavenger receptor with a role in the anti-inflammatory response.. Ann Med.

[45] Weaver LK, Hintz-Goldstein KA, Pioli PA, Wardwell K, Qureshi N (2006). Pivotal advance: activation of cell surface Toll-like receptors causes shedding of the hemoglobin scavenger receptor CD163.. J Leukoc Biol.

[46] Cordone I, Matutes E, Catovsky D (1992). Characterisation of normal peripheral blood cells in cycle identified by monoclonal antibody Ki-67.. J Clin Pathol.

[47] Goud TJ, van Furth R (1975). Proliferative characteristics of monoblasts grown in vitro.. J Exp Med.

[48] van Furth R, Raeburn JA, van Zwet TL (1979). Characteristics of human mononuclear phagocytes.. Blood.

[49] Gerdes J, Lemke H, Baisch H, Wacker HH, Schwab U (1984). Cell cycle analysis of a cell proliferation-associated human nuclear antigen defined by the monoclonal antibody Ki-67.. J Immunol.

[50] Passlick B, Flieger D, Ziegler-Heitbrock HW (1989). Identification and characterization of a novel monocyte subpopulation in human peripheral blood.. Blood.

[51] Marcondes MC, Lanigan CM, Burdo TH, Watry DD, Fox HS (2008). Increased expression of monocyte CD44v6 correlates with the deveopment of encephalitis in rhesus macaques infected with simian immunodeficiency virus.. J Infect Dis.

[52] Brenchley JM, Price DA, Schacker TW, Asher TE, Silvestri G (2006). Microbial translocation is a cause of systemic immune activation in chronic HIV infection.. Nat Med.

[53] Ancuta P, Kamat A, Kunstman KJ, Kim EY, Autissier P (2008). Microbial translocation is associated with increased monocyte activation and dementia in AIDS patients.. PLoS ONE.

[54] Fabriek BO, Van Haastert ES, Galea I, Polfliet MM, Dopp ED (2005). CD163-positive perivascular macrophages in the human CNS express molecules for antigen recognition and presentation.. Glia.

[55] Weaver LK, Pioli PA, Wardwell K, Vogel SN, Guyre PM (2007). Up-regulation of human monocyte CD163 upon activation of cell-surface Toll-like receptors.. J Leukoc Biol.

[56] Bruck W, Porada P, Poser S, Rieckmann P, Hanefeld F (1995). Monocyte/macrophage differentiation in early multiple sclerosis lesions.. Ann Neurol.

[57] Otani I, Mori K, Sata T, Terao K, Doi K (1999). Accumulation of MAC387+ macrophages in paracortical areas of lymph nodes in rhesus monkeys acutely infected with simian immunodeficiency virus.. Microbes Infect.

[58] Ragin AB, Wu Y, Storey P, Cohen BA, Edelman RR (2006). Bone marrow diffusion measures correlate with dementia severity in HIV patients.. AJNR Am J Neuroradiol.

[59] Westmoreland SV, Halpern E, Lackner AA (1998). Simian immunodeficiency virus encephalitis in rhesus macaques is associated with rapid disease progression.. J Neurovirol.

[60] Moller HJ, de Fost M, Aerts H, Hollak C, Moestrup SK (2004). Plasma level of the macrophage-derived soluble CD163 is increased and positively correlates with severity in Gaucher's disease.. Eur J Haematol.

[61] Giri MS, Nebozyhn M, Raymond A, Gekonge B, Hancock A (2009). Circulating monocytes in HIV-1-infected viremic subjects exhibit an antiapoptosis gene signature and virus- and host-mediated apoptosis resistance.. J Immunol.

[62] Serbina NV, Pamer EG (2006). Monocyte emigration from bone marrow during bacterial infection requires signals mediated by chemokine receptor CCR2.. Nat Immunol.

[63] Tsou CL, Peters W, Si Y, Slaymaker S, Aslanian AM (2007). Critical roles for CCR2 and MCP-3 in monocyte mobilization from bone marrow and recruitment to inflammatory sites.. J Clin Invest.

[64] Lapidot T, Petit I (2002). Current understanding of stem cell mobilization: the roles of chemokines, proteolytic enzymes, adhesion molecules, cytokines, and stromal cells.. Exp Hematol.

[65] Peled A, Petit I, Kollet O, Magid M, Ponomaryov T (1999). Dependence of human stem cell engraftment and repopulation of NOD/SCID mice on CXCR4.. Science.

[66] Ancuta P, Liu KY, Misra V, Wacleche VS, Gosselin A (2009). Transcriptional profiling reveals developmental relationship and distinct biological functions of CD16+ and CD16- monocyte subsets.. BMC Genomics.

[67] Komohara Y, Hirahara J, Horikawa T, Kawamura K, Kiyota E (2006). AM-3K, an anti-macrophage antibody, recognizes CD163, a molecule associated with an anti-inflammatory macrophage phenotype.. J Histochem Cytochem.

[68] Sasseville VG, Lackner AA (1997). Neuropathogenesis of simian immunodeficiency virus infection in macaque monkeys.. J Neurovirol.

[69] Nath A (1999). Pathobiology of human immunodeficiency virus dementia.. Semin Neurol.

[70] Bell JE (1998). The neuropathology of adult HIV infection.. Rev Neurol (Paris).

[71] Lifson JD, Rossio JL, Piatak M, Parks T, Li L (2001). Role of CD8(+) lymphocytes in control of simian immunodeficiency virus infection and resistance to rechallenge after transient early antiretroviral treatment.. J Virol.

